# Regulation and Prognostic Relevance of Symmetric Dimethylarginine Serum Concentrations in Critical Illness and Sepsis

**DOI:** 10.1155/2013/413826

**Published:** 2013-06-27

**Authors:** Alexander Koch, Ralf Weiskirchen, Jan Bruensing, Hanna Dückers, Lukas Buendgens, Julian Kunze, Michael Matthes, Tom Luedde, Christian Trautwein, Frank Tacke

**Affiliations:** ^1^Department of Medicine III, RWTH University Hospital Aachen, Pauwelsstraße 30, 52074 Aachen, Germany; ^2^Institute of Clinical Chemistry and Pathobiochemistry, RWTH University Hospital Aachen, Pauwelsstraße 30, 52074 Aachen, Germany

## Abstract

In systemic inflammation and sepsis, endothelial activation and microvascular dysfunction are characteristic features that promote multiorgan failure. As symmetric dimethylarginine (SDMA) impacts vascular tension and integrity via modulating nitric oxide (NO) pathways, we investigated circulating SDMA in critical illness and sepsis. 247 critically ill patients (160 with sepsis, 87 without sepsis) were studied prospectively upon admission to the medical intensive care unit (ICU) and on day 7, in comparison to 84 healthy controls. SDMA serum levels were significantly elevated in critically ill patients at admission to ICU compared to controls and remained stably elevated during the first week of ICU treatment. The highest SDMA levels were found in patients with sepsis. SDMA levels closely correlated with disease severity scores, biomarkers of inflammation, and organ failure (renal, hepatic, and circulatory). We identified SDMA serum concentrations at admission as an independent prognostic biomarker in critically ill patients not only for short-term mortality at the ICU but also for unfavourable long-term survival. Thus, the significant increase of circulating SDMA in critically ill patients indicates a potential pathogenic involvement in endothelial dysfunction during sepsis and may be useful for mortality risk stratification at the ICU.

## 1. Introduction

Alterations in microvascular perfusion are common characteristics of patients with systemic inflammation and sepsis and substantially contribute to the development of organ failure [1, 2]. Microcirculatory defects in critically ill patients such as capillary leakage and disturbed capillary perfusion are not necessarily reflected by macrohemodynamic parameters (e.g., mean arterial blood pressure, cardiac index, and central venous oxygen saturation) that are commonly assessed at the intensive care unit (ICU) [3]. In fact, a recent study revealed that although global hemodynamic variables were relatively preserved in patients with severe sepsis, their microvascular perfusion as assessed by complex invasive flow imaging techniques was severely altered, predicted the progression of organ failure and the overall mortality risk [2]. The underlying mechanisms of microvascular dysfunction in sepsis result from different factors such as endothelial dysfunction, leukocyte-endothelium interactions, coagulation and inflammatory disorders, hemorheologic abnormalities, and functional shunting [4]. 

The activation of the endothelium, as reflected by increased levels of circulating biomarkers, has been suggested as a main promoter in the pathogenesis of disturbed microcirculation [5]. Based on the potent vasodilative effects of nitric oxide (NO), the arginine-NO pathway might be substantially involved in inflammation, infection, and organ injury [6]. The natural inhibitor of NO synthase, asymmetric dimethylarginine (ADMA), has been found elevated in patients with sepsis and related to mortality risk [7–11]. ADMA is assumed to exert detrimental effects on endothelial function, cardiovascular homeostasis, and cardiovascular outcomes. In contrast, relatively little is known about the other methylated form of L-arginine, symmetric dimethylarginine (SDMA) [12]. 

SDMA is generated as the isomer form of ADMA by protein hydrolysis [13]. Unlike ADMA, SDMA is not a direct inhibitor of NO synthase [14]. Thus, SDMA has long been regarded as an inert, functionally inactive molecule. However, using highly specific *in vitro* models with primary endothelial cells, SDMA was found to reduce endothelial NO synthesis via competition with arginine at the cellular transporter and increased intracellular reactive oxygen species in a dose-dependent manner, already at very low, “physiological” concentrations [15]. Circulating levels of SDMA in serum have been consecutively investigated in several cohorts of patients with cardiovascular and renal diseases, demonstrating an association of SDMA with glomerular filtration rate and extent of coronary artery disease and atherosclerosis [12].

We hypothesized that SDMA might be involved in endothelial dysfunction during critical illness and sepsis, resulting in organ failure. Therefore, we investigated SDMA serum levels in a large cohort of 247 consecutively enrolled critically ill patients in order to identify associations between SDMA and organ dysfunction, metabolism and disease severity as well as to assess the prognostic value of SDMA for ICU and long-term mortality.

## 2. Material and Methods

### 2.1. Study Design and Patient Characteristics

All patients that were admitted to the medical ICU were consecutively enrolled, except for patients who were expected to have a short-term (<72 h) intensive care treatment due to postinterventional observation or acute intoxication [16]. Patient data and blood samples were collected prospectively. Patients who met the criteria proposed by the American College of Chest Physicians and the Society of Critical Care Medicine Consensus Conference Committee for severe sepsis and septic shock were categorized as sepsis patients and the others as nonsepsis patients [17]. After discharge from our hospital, the outcome was assessed during a follow-up period by directly contacting the patients, their relatives, or primary care physician. The study protocol was approved by the local ethics committee (EK 150/06). Written informed consent was obtained from the patient, his or her spouse, or the appointed legal guardian.

As a control population, we analyzed 84 healthy blood donors (57 male, 27 female) with normal values for blood counts, C-reactive protein, and liver enzymes. 

### 2.2. SDMA Measurements

Blood samples were collected upon admission to the ICU (prior to therapeutic interventions) as well as in the morning of day 7 after admission. After centrifugation at 4°C for 10 minutes, serum and plasma aliquots of 1 mL were frozen immediately at –80°C. SDMA serum concentrations were analysed using a commercial enzyme immunoassay (Immundiagnostik, Bensheim, Germany). The scientist performing experimental measurements was fully blinded to any clinical or other laboratory data of the patients or controls. Due to limited technical resources and changes in the original patient cohort due to discharges from the ICU and deaths, follow-up SDMA measurements were only performed in 42 patients.

### 2.3. Statistical Analysis

Due to the skewed distribution of most of the parameters, data are presented as median and range. Differences between two groups were assessed by Mann-Whitney *U* test, and multiple comparisons between more than two groups have been conducted by Kruskal-Wallis ANOVA and Mann-Whitney *U* test for post hoc analysis. Box plot graphics illustrate comparisons between subgroups, and they display a statistical summary of the median, quartiles, range, and extreme values. The whiskers extend from the minimum to the maximum values excluding outside and far out values which are displayed as separate points. An outside value (indicated by an open circle) was defined as a value that is smaller than the lower quartile minus 1.5-times interquartile range or larger than the upper quartile plus 1.5 times the interquartile range. A far out value was defined as a value that is smaller than the lower quartile minus three times interquartile range or larger than the upper quartile plus three times the interquartile range [18]. All values, including “outliers,” have been included for statistical analyses. 

Correlations between variables have been analysed using the Spearman correlation tests. Single parameters that correlated significantly with SDMA levels at admission were included in a multivariate linear regression analysis with SDMA as the dependent variable to identify independent predictors of elevated SDMA. The prognostic value of the variables was tested by univariate and multivariate analyses in the Cox regression model. Kaplan-Meier curves were plotted to display the impact on survival [19]. *P* values below 0.05 were considered statistically significant. Statistical analyses were performed with SPSS (SPSS, Chicago, IL, USA).

## 3. Results

### 3.1. SDMA Serum Levels Are Significantly Elevated in Critically Ill Patients, Especially in Conditions of Sepsis

**Figure 1:**
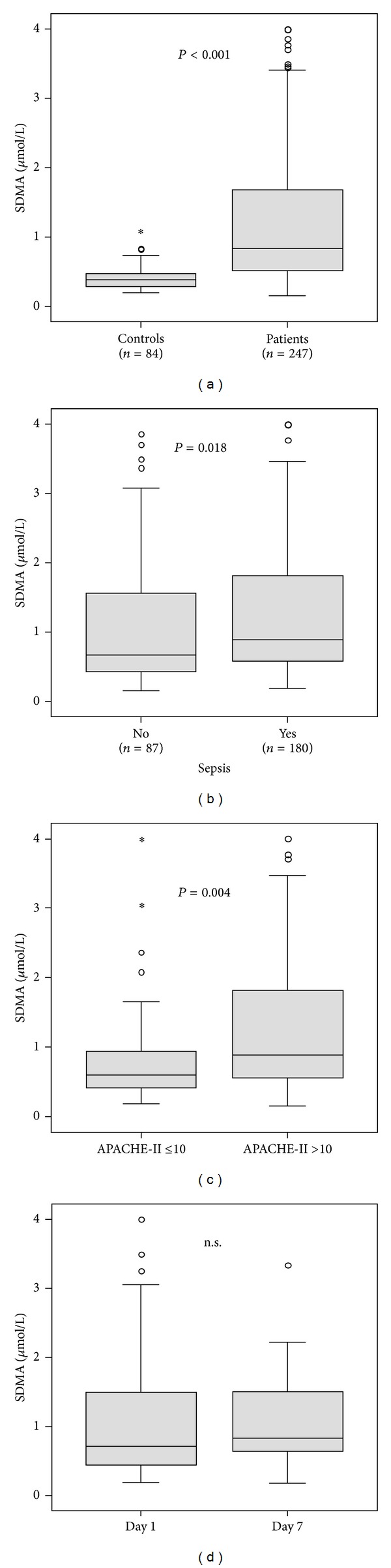
Serum SDMA concentrations in critically ill patients. (a) At admission to the medical ICU, serum SDMA levels were significantly (*P* < 0.001, *U* test) elevated in critically ill patients (*n* = 247) as compared to healthy controls (*n* = 84). (b) SDMA serum levels at ICU admission were significantly increased in ICU patients with sepsis (*n* = 180) compared to patients without sepsis (*n* = 87). (c) SDMA serum levels at ICU admission were significantly increased in ICU patients with higher degree of disease severity, as displayed by the APACHE-II score. (d) In 42 patients, SDMA levels were measured at admission (day 1) and at 1 week (day 7) of ICU therapy. SDMA levels remained stable during the first week of ICU treatment (paired Wilcoxon test).

In order to investigate SDMA in critical illness, we measured SDMA serum concentrations in a large cohort of medical ICU patients at admission (= before therapeutic intervention) and on day 7 (Table 1). SDMA serum levels were significantly higher in ICU patients (*n* = 247, median 0.84 *μ*mol/L, and range 0.15–4.0) as compared with healthy controls (*n* = 84, median 0.38 *μ*mol/L, range 0.20–1.06, and *P* < 0.001; Figure 1(a)). No associations between SDMA levels and sex or age were observed in controls (data not shown).

About two thirds (*n* = 160) of the ICU patients consecutively enrolled into our study presented with either sepsis or septic shock upon ICU admission (Table 2). Importantly, patients with sepsis (*n* = 160, median 0.89 *μ*mol/L, and range 0.19–4.0) had significantly higher SDMA serum concentrations at ICU admission compared to patients with non-septic origin of critical illness (*n* = 87, median 0.67 *μ*mol/L, range 0.15–3.86, Figure 1(b)). The site of infection (Table 2) was not associated with SDMA levels (detailed data not shown). However, SDMA levels were related to disease severity, as patients with APACHE-II score values greater than 10 displayed significantly elevated SDMA serum concentrations (Figure 1(c)).

Elevated SDMA levels had been observed in patients with metabolic and cardiovascular disorders [12]. In our cohort of critically ill patients, SDMA levels did not differ between patients with or without type 2 diabetes or obesity, defined as a body mass index >30 kg/m² (detailed data not shown).

In 42 patients, paired blood samples were available for SDMA measurements at ICU admission and at day 7 of ICU treatment. Individual SDMA levels remained stable during the first week of ICU therapy (Table 1, Figure 1(d), not significant by paired Wilcoxon test).

### 3.2. SDMA Serum Concentrations at Admission to the ICU Are Closely Correlated to Organ Function, Inflammation, Metabolism, and Disease Severity

**Figure 2:**
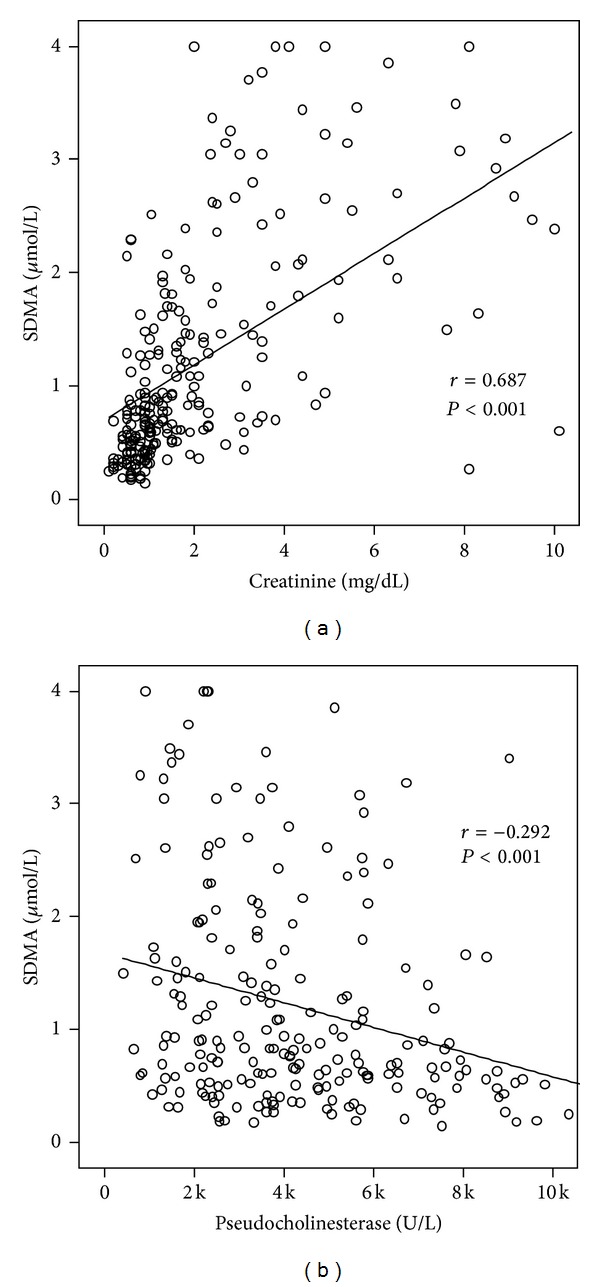
Serum SDMA concentrations in critically ill patients are correlated with renal and hepatic organ failure. Serum ADMA levels were measured in *n* = 247 critically ill patients at admission to the ICU. Serum SDMA correlated significantly with renal failure (creatinine, (a)) or hepatic failure (pseudocholinesterase, (b)). Spearman rank correlation test, *r* and *P* values are given in the figure.

In order to understand possible mechanisms underlying elevated serum SDMA levels in critically ill patients, we performed extensive correlation analyses with various laboratory parameters. At admission to the ICU, serum SDMA concentrations were closely correlated to biomarkers displaying organ dysfunction. In detail, SDMA was found to correlate significantly with markers reflecting renal failure such as creatinine (*r* = 0.687, *P* < 0.001), cystatin C (*r* = 0.714, *P* < 0.001) or inversely with their glomerular filtration rates (Figure 2(a)). Moreover, SDMA levels correlated with clinically used biomarkers of hepatic dysfunction like reduced protein (*r* = −0.172, *P* = 0.013), pseudocholinesterase (*r* = −0.292, *P* < 0.001), or bilirubin excretion (Figure 2(b)). 

In line with the elevated SDMA concentrations observed in patients with sepsis, various biomarkers indicating systemic inflammation were associated with circulating SDMA. In fact, SDMA levels correlated with white blood cell counts (0.190, *P* = 0.003), C-reactive protein (*r* = 0.261, *P* < 0.001), procalcitonin (*r* = 0.407, *P* < 0.001), tumor necrosis factor (*r* = 0.324, *P* = 0.004), and soluble urokinase plasminogen activator receptor (suPAR, *r* = 0.494, *P* < 0.001), a prognostic biomarker in sepsis [20]. SDMA levels correlated with ADMA (*r* = 0.384, *P* < 0.001) as well [11].

When selected parameters that were correlated with SDMA serum levels by univariate analysis (i.e., creatinine, pseudocholinesterase, bilirubin, suPAR, ADMA, C-reactive protein, and procalcitonin) were included in a multivariate regression analysis, only creatinine (*P* < 0.001) and procalcitonin (*P* = 0.023), but not liver function markers, ADMA, or suPAR, remained independent predictors of SDMA concentrations (Table 3).

### 3.3. SDMA Serum Levels Are an Independent Prognostic Biomarker for ICU and Overall Long-Term Mortality in Critically Ill Patients

**Figure 3:**
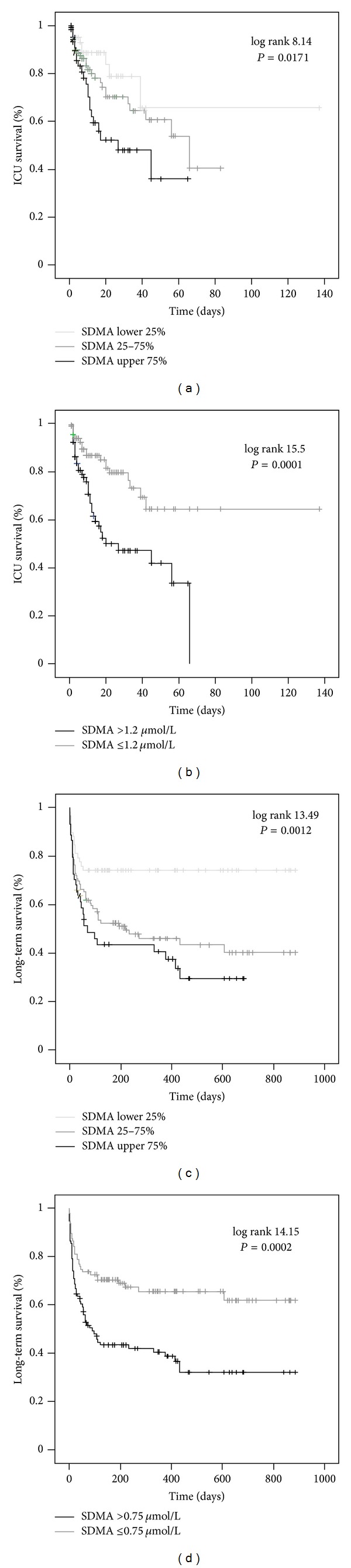
Prediction of mortality by SDMA serum concentrations. ((a)-(b)) Kaplan-Meier survival curves of ICU patients are displayed, showing that patients with SDMA levels of upper quartile (on admission >1.7 *μ*mol/L; black (a)) had an increased short-term mortality at the ICU as compared to patients with ADMA serum concentrations of lower quartile (on admission <0.51 *μ*mol/L; light grey) or middle 50% (grey). Best discrimination between ICU survivors and nonsurvivors was achieved with an SDMA cutoff value of 1.2 *μ*mol/L (b). Log rank and *P* values are given in the figure. ((c)-(d)) Kaplan-Meier survival curves of ICU patients are displayed, showing that patients with SDMA levels of upper quartile (on admission >1.7 *μ*mol/L; black (c)) had an increased long-term mortality at the ICU as compared to patients with SDMA serum concentrations of lower quartile (on admission <0.51 *μ*mol/L; light grey) or middle 50% (grey). Best discrimination between overall survivors and nonsurvivors was achieved with an ADMA cutoff value of 0.75 *μ*mol/L (d).

Based on the close correlation between SDMA levels at admission and disease severity scores, we hypothesized that circulating SDMA might be capable of identifying patients at high risk of mortality. Indeed, patients that died during the course of ICU treatment (about one quarter of the total cohort) had significantly higher serum SDMA levels at admission compared with the ICU survivors (median 1.33 versus 0.74 *μ*mol/L, *P* = 0.001). We thus performed Cox regression analyses and Kaplan-Meier curves to assess the impact of the initial SDMA serum concentrations on ICU mortality among critically ill patients. Low SDMA levels upon admission to the ICU were a strong prognostic predictor for ICU survival (*P* = 0.021, Cox regression analyses). In multivariate Cox regression analyses, including markers of inflammation/infection (CRP, WBC), circulatory (lactate), hepatic (bilirubin, protein, and INR), and renal (creatinine) deterioration at admission, SDMA remained an independent significant prognostic parameter (hazard ratios and *P* values are presented in Table 4). Kaplan-Meier curves displayed that patients with SDMA levels of the upper quartile (>1.7 *μ*mol/L) had the highest mortality (log rank 8.14, *P* = 0.0171, Figure 3(a)). We found the best cutoff value to discriminate survivors from non-ICU survivors for serum SDMA of 1.2 *μ*mol/L (log rank 15.15, *P* = 0.0001, Figure 3(b)). 

During the follow-up observation period of approximately three years, the overall case fatality rate increased to 47.3% of the study cohort (Table 1). SDMA serum concentrations at admission to the ICU were significantly higher in patients with unfavourable outcome (median 1.09 versus 0.67 *μ*mol/L, *P* < 0.001). By Cox regression analysis, initial serum SDMA levels significantly predicted long-term prognosis (*P* = 0.010). The prognostic value remained significant also by multivariate analysis (Table 5). Kaplan-Meier curves proved that SDMA levels of the highest quartile (>1.7 *μ*mol/L) were strongly associated with fatal outcome (log rank test 13.49, *P* = 0.0012, Figure 3(c)). SDMA levels of 0.75 *μ*mol/L discriminated the long-term prognosis of critically ill patients (log rank test 14.15, *P* = 0.0002, Figure 3(d)). Interestingly, when SDMA levels were adjusted to renal function by calculating the SDMA/creatinine ratio, patients that died during the observation period still displayed significantly elevated SDMA/creatinine values (*P* = 0.012, detailed data not shown), confirming that the association of SDMA with long-term mortality was independent of renal function. 

## 4. Discussion

The excessive endothelial activation in systemic inflammation and sepsis affects hemostasis, leukocyte trafficking, vascular permeability, and the extent of disturbed microcirculation [5]. There is experimental and clinical evidence that dysregulation of the arginine-NO pathway critically contributes to this process [21]. It had been previously demonstrated that ADMA as an endogenous NO synthase inhibitor is a promoter of vascular dysfunction in patients with sepsis [7–10]. Our study now shows that also SDMA, another methylated form of L-arginine, is also significantly upregulated in critically ill patients, especially in conditions of sepsis, associated with inflammation and organ failure as well as a yet unrecognized indicator for mortality risk in medical ICU patients.

A prominent finding in our heterogeneous cohort of critically ill medical patients was the independent association of SDMA serum levels with biomarkers reflecting renal dysfunction and systemic inflammation by multivariate analyses. The fact that renal function was an important independent determinant of circulating SDMA levels was not surprising, because SDMA is excreted via the urine, and SDMA has been found elevated in studies of patients with end-stage renal disease [12, 22]. There is also experimental evidence that dimethylarginines can be metabolized by the liver as well [23], which would well explain its increase in ICU patients with hepatic dysfunction. Furthermore, the close correlation between SDMA levels and inflammatory biomarkers such as procalcitonin or tumor necrosis factor may indicate that protein catabolism induced by systemic inflammation might contribute to elevated systemic SDMA levels in critically ill patients. Due to our study design, which focussed on regulation of SDMA in critically ill patients at admission to the ICU, we were unable to further analyse whether the persistence of elevated SDMA, as observed in patients with available longitudinal SDMA measurements, reflects persistent systemic inflammation or is rather an epiphenomenon of multiorgan failure in these patients. 

Our study identified SDMA as a prognostic marker in patients with critical illness, both for ICU and long-term mortality. Importantly, SDMA remained independently associated with mortality in multivariate regression analyses, corroborating that SDMA is not only an epiphenomenon of acute organ dysfunction. These data strongly indicate that elevated SDMA levels in ICU patients reflect prognostically relevant pathomechanisms such as microcirculatory dysfunction due to endothelial activation. The accumulation of SDMA might reduce endothelial NO synthesis, as it competes with arginine for cellular transport across the *y* ^+^ transporter and might promote endothelial stress, as it has been showed to increase intracellular reactive oxygen species in human endothelium [12, 15]. Similar cause-effect relationships have been proposed for chronic, “low-grade inflammatory” processes such as atherosclerosis [24]. One might speculate whether therapeutic interventions intended to increase vascular tension during the hyperdynamic state of sepsis via modulating arginine-NO interactions could be beneficial in critically ill patients [25].

Despite its potential pathogenic implications, SDMA serum levels were closely associated with ICU as well as long-term mortality risk in our cohort of critically ill medical patients. Our study now identified possible cutoff values of circulating SDMA as indicators for increased mortality risk. This raises the possibility that implementing SDMA in risk stratification algorithms might further increase the prognostic accuracy of current clinical scoring system at the ICU. Future studies should therefore not only aim at exploring the pathogenic role of SDMA in sepsis and concomitant endothelial dysfunction but also evaluate the clinical applicability of SDMA measurements as a prognostic biomarker in critical illness.

## 5. Conclusions

Our study demonstrates significantly upregulated serum levels of SDMA in critically ill patients, especially in patients with sepsis. The potential value of SDMA as an indicator of endothelial dysfunction in medical ICU patients and its correlations to biomarkers of renal, liver, and circulatory failure function should be confirmed in experimental models of systemic inflammation and in different clinical settings. The clear association of circulating SDMA levels with clinically relevant endpoints such as ICU or long-term mortality gives rise to the expectation that integrating SDMA into current tools of risk assessment in critically ill patients might improve their prognostic accuracy.

## Figures and Tables

**Table 1:** Baseline patient characteristics and SDMA serum measurements.

Parameter	All patients	Sepsis	Nonsepsis	*P* value
Number	247	160	87	—
Sex (male/female)	145/102	94/66	51/36	—
Age median (range) [years]	63 (18–90)	64 (20–90)	60 (18–85)	n.s.
APACHE-II score median (range)	17 (2–43)	19 (3–43)	15 (2–33)	0.002
SOFA score median (range)	9 (0–19)	11 (3–19)	7 (0–16)	<0.001
ICU days median (range)	8 (1–137)	10 (1–137)	6 (1–45)	<0.001
Death during ICU *n* (%)	60 (24.3)	45 (28.1)	15 (17.2)	—
Death during follow-up *n* (%)	115 (47.3)	85 (54.1)	30 (34.9)	—
Mechanical ventilation *n* (%)	171 (71.5)	117 (75.5)	54 (64.3)	—
Ventilation time median (range) [h]	126 (0–2966)	181 (0–2966)	63 (0–986)	0.019
Preexisting diabetes *n* (%)	72 (29.1)	45 (28.1)	27 (31.0)	—
BMI median (range) [m²/kg]	25.8 (15.9–86.5)	25.8 (17.1–86.5)	25.4 (15.9–53.3)	n.s.
WBC median (range) [×10³/*µ*L]	12.7 (0.1–149)	13.8 (0.1–149)	11.4 (1.8–29.6)	0.010
CRP median (range) [mg/dL]	103 (5–230)	162 (5–230)	17 (5–230)	<0.001
Procalcitonin median (range) [*µ*g/L]	1.0 (0.05–248)	3.2 (0.1–248)	0.24 (0.05–100)	<0.001
Creatinine median (range) [mg/dL]	1.3 (0.1–21.6)	1.6 (0.1–21.6)	1.0 (0.2–11.5)	n.s.
GFR Cystatin median (range) [mL/min]	33 (3–379)	27 (3–379)	58 (5–379)	0.013
INR median (range)	1.17 (0.9–4.64)	1.18 (0.92–4.64)	1.16 (0.9–4.32)	n.s.
SDMA day 1 median (range) [*µ*mol/L]	0.84 (0.15–4.0)	0.89 (0.19–4.0)	0.67 (0.15–3.86)	0.018
SDMA day 7 median (range) [*µ*mol/L]	0.82 (0.18–3.34)	0.85 (0.18–3.34)	0.81 (0.23–1.50)	n.s.

For quantitative variables, median and range (in parenthesis) are given. Differences between sepsis and nonsepsis patients were tested for significance (*P* values [*U* test] are given in the table). APACHE: Acute Physiology and Chronic Health Evaluation; BMI: body mass index; CRP: C-reactive protein; ICU: intensive care unit; INR: international normalized ratio; SDMA: symmetric dimethylarginine; SOFA: sequential organ failure assessment; WBC: white blood cell count.

**Table 2:** Disease etiology of the study population.

	Sepsis	Nonsepsis
	*n* = 160
Etiology of sepsis critical illness *n* (%)		
Site of infection		
Pulmonary	91 (56.9)	
Abdominal	29 (18.1)	
Urogenital	14 (8.8)	
Other	26 (16.2)	

Etiology of nonsepsis critical illness *n* (%)		
Cardiopulmonary disorder		35 (40.2)
Acute pancreatitis		11 (12.6)
Decompensated liver cirrhosis		16 (18.4)
Severe gastrointestinal hemorrhage		7 (8.0)
Non-sepsis other		18 (20.7)

**Table 3:** Multivariate regression analysis of parameters determining SDMA levels.

Parameter	Standardized coefficient beta	*t*-value	*P* value
Creatinine	0.502	7.108	<0.001
Procalcitonin	0.164	2.311	0.023
suPAR	0.151	1.587	NS
C-reactive protein	0.019	0.258	NS
Pseudocholinesterase	−0.089	−1.112	NS
Bilirubin	0.003	0.042	NS
ADMA	0.109	1.378	NS

ADMA: asymmetric dimethylarginine; SDMA: symmetric dimethylarginine; suPAR: soluble urokinase plasminogen activator receptor.

**Table 4:** Uni- and multivariate Cox regression analyses for SDMA levels at admission to predict ICU mortality.

Parameter	Unadjusted HR (95%-CI)	*P* value	Adjusted HR (95%-CI)	*P* value
SDMA	1.349 (1.059–1.719)	0.015	1.379 (1.012–1.879)	0.042
Protein	0.961 (0.939–0.984)	0.001	0.966 (0.940–0.993)	0.012
INR	1.890 (1.370–2.607)	<0.001	1.537 (1.004–2.354)	0.048
Lactate	1.169 (1.085–1.260)	<0.001	1.142 (1.058–1.231)	0.001
Creatinine	1.026 (0.937–1.123)	NS	—	NS
White blood cell count	0.986 (0.955–1.018)	NS	—	NS
C-reactive protein	1.0 (0.997–1.003)	NS	—	NS

**Table 5:** Uni- and multivariate Cox regression analyses for SDMA levels at admission to predict overall mortality.

Parameter	Unadjusted HR (95%-CI)	*P* value	Adjusted HR (95%-CI)	*P* value
SDMA	1.275 (1.067–1.524)	0.007	1.357 (1.088–1.692)	0.007
Protein	0.965 (0.948–0.982)	<0.001	0.973 (0.954–0.992)	0.006
INR	1.630 (1.226–2.168)	0.001	1.410 (0.997–1.994)	0.05
Lactate	1.133 (1.054–1.217)	0.001	1.122 (1.042–1.207)	0.002
Creatinine	1.009 (0.944–1.079)	NS	—	NS
White blood cell count	0.989 (0.967–1.012)	NS	—	NS
C-reactive protein	1.001 (0.999–1.004)	NS	—	NS
